# Retinal-Image Quality and Night-Vision Performance after Alcohol Consumption

**DOI:** 10.1155/2014/704823

**Published:** 2014-04-07

**Authors:** José J. Castro, Antonio M. Pozo, Manuel Rubiño, Rosario G. Anera, Luis Jiménez del Barco

**Affiliations:** Laboratory of Vision Sciences and Applications, Department of Optics, University of Granada, Mecenas Building (Sciences Faculty), Avenida de Fuentenueva, s/n. 18071 Granada, Spain

## Abstract

*Purpose.* To evaluate the influence of alcohol consumption on the retinal-image quality and visual performance under surrounding low-illumination conditions. *Methods.* A volunteer sample of 67 subjects was analyzed. Optical quality of the eye was evaluated by means of the Strehl ratio, the Objective Scattering Index (OSI), and the tear-film quality. We used the visual disturbance index (VDI) to evaluate visual performance under low-illumination conditions and we measured the pupil size under these conditions. The tear-film volume was also measured. All measurements were made before and after alcohol consumption and patients were classified into two groups depending on their breath alcohol content (BrAC): low-alcohol (BrAC < 0.25 mg/L) and high-alcohol content (BrAC ≥ 0.25 mg/L). *Results.* The VDI was significantly higher after alcohol consumption: the higher the BrAC, the higher the deterioration of the visual discrimination capacity. The pupil size increased significantly for the high-BrAC group. Parameters evaluating optical quality deteriorated after alcohol consumption. *Conclusion.* The visual performance under low-illumination conditions and the retinal-image quality were deteriorated after alcohol consumption, especially for the high-alcohol group. Furthermore, some physiological changes were observed under effects for high-alcohol contents, such as an increase in the pupil size and disturbances in the tear film, which deteriorated optical quality.

## 1. Introduction

The optical quality of the human eye has been widely studied in recent years, as well as its influence on the visual performance. This optical quality depends on the state of the ocular media [1–3]. Some external factors also can have an influence on optical quality, such as pupil dilation under low-illumination conditions, where the intraocular scattering and ocular aberrations are expected to augment, contributing to the deterioration of the retinal-image quality [4, 5]. All these aspects can interfere with visual performance, as previous studies have demonstrated [1–3, 6], but also consumption of some types of substance, such as alcohol, can have a negative impact on visual functions [7, 8], hampering many of the daily tasks that require keen vision and full visual performance, such as driving [9], especially under night-time conditions. The World Health Organization (WHO) lists alcohol as one of the main causes of traffic accidents [10, 11].

Several works have shown the impact of alcohol intake on different visual functions reporting a deterioration in visual function after alcohol consumption [7, 8, 12, 13]. Other authors have found physiological changes [14, 15]. However, no studies are available concerning the influence of alcohol intake on the retinal-image quality and the visual performance under low-illumination conditions. Visual performance can be evaluated by means of the visual-discrimination capacity, which is impaired by visual disturbances perceived by the observer. This impairment could be intensified by alcohol intake. Low levels of illuminance together with alcohol consumption could be two important factors that could limit the visual abilities of the subject.

In the present work, we study the influence of alcohol consumption on the retinal-image quality and visual performance under low-illumination conditions. For this, we use a double-pass device for an objective measurement of ocular optical quality, a pupillometer to measure the pupil diameter, and a tear-film test to quantify the tear volume of the eye. Visual performance under low-illuminance levels is characterized using the visual-discrimination capacity, which is useful to gain a complete characterization of visual performance by comparing results before and after alcohol consumption.

## 2. Methods

### 2.1. Participants

A total of 67 subjects (32 males, 35 females) participated in the study (134 eyes to be studied), with a mean age of 27.6 ± 8.1 years. Admission criteria for the subjects were that all observers had to be moderate social drinkers older than 18 years old, but without being under any pharmacological treatment. They had to reach a corrected monocular VA ≥ 1.0 in both eyes and have no pathological conditions that could affect visual performance. All participants in the experiments gave their informed consent in accordance with the Helsinki Declaration. A battery of tests was performed under two conditions: before and after having alcoholic drinks.

### 2.2. Measuring Breath Alcohol Content

The ethanol concentration for each participant was determined by measuring the breath alcohol content (BrAC), expressed in milligrams of ethanol per litre of exhaled air (mg/L), using a breath analyser [16]. We used the Dräger Alcotest 7110 MK-III (Dräger Safety AG & Co. KGaA. Lübeck, Germany), previously calibrated. This instrument is an evidential breath-alcohol analyser and is used in countries such as Spain for legal purposes and for traffic controls. Ethanol was administered orally by inviting participants to consume an alcoholic beverage (two or more glasses of red wine), so that different rates of alcohol were found. The red wine used was* Ribera del Farbes* (Pago De Almaraes wineries, S.L. Benalúa de Guadix, Granada, Spain), a young red with 13.5% of alcohol content. The participants were asked to consume these drinks within a 60 min period. After this period, three measurements of BrAC were made with the breath analyser, every 30 min. For each participant, we calculated the mean BrAC, and then, participants were assigned to two groups: a low-BrAC group with BrAC < 0.25 mg/L and a high-BrAC group with BrAC ≥ 0.25 mg/L (Table 1). This classification was made taking into account drinking and driving laws in most European countries, following the recommendations of the WHO [10, 11].

### 2.3. Retinal-Image Quality

To determine the optical quality of the eye, we took objective data from an optical device based on the double-pass technique [17, 18]. We used the commercial device OQAS (Optical Quality Analysis System, Visiometrics S.L. Tarrasa, Spain), which provides data on ocular aberrations and scattering. To evaluate the optical quality, we took the Strehl ratio and the Objective Scatter Index (OSI). The Strehl ratio ranges from 0 to 1 and is defined as the ratio between the 2D-Modulation Transfer Function (2D-MTF) area of the eye and the diffraction-limited 2D-MTF area. A lower value of this parameter indicates a greater contribution of aberrations and ocular scattering and therefore poorer optical quality. We also took the OSI, a parameter that permits the objective quantification of the intraocular scattering. For younger eyes, the OSI value is around 0.5, while for eyes with a cataract the value is higher than 4 [19]. Furthermore, to evaluate the optical quality of the tear film, we traced the time course of the OSI. For that, we measured the OSI in 0.5-seconds steps, from 0 to 10 seconds (without blinking for all the 10-sec measurement). We made OQAS measurements before and after alcohol consumption, in a dark room. The data from the double-pass device were performed for a 4 mm pupil.

### 2.4. Tear-Film Test

To measure the tear-film volume, a phenol red thread (PRT) tear test was used, the Zone-Quick (Menicon. Tokyo, Japan) consisting of a cotton thread treated with the pH indicator. The folded 3 mm portion of the thread is placed on the palpebral conjunctiva, and, after 15 seconds, the thread is removed. The length of the red portion of the thread is measured in millimeters and is a quantification of the tear-film volume: the greater the length, the higher the tear-film volume. For each participant, before and after alcohol consumption, the PRT test was performed individually for both eyes after OQAS measurements.

### 2.5. Visual-Discrimination Capacity and Pupil Size

To evaluate the visual performance under surrounding low-illumination conditions, we quantified the visual disturbances perceived by the subject using a visual test conducted by the software Halo v1.0 [3, 6]. The subject's task consisted of detecting luminous peripheral stimuli around a central high-luminance stimulus over a dark background. All of the stimuli were achromatic. The distance from the observer to the test monitor (1024 × 768  pixels LCD monitor) was 2.5 m and the test was performed monocularly, with best correction. The size of the stimuli was 30 pixels for the radius of the central stimulus and 1 pixel for the peripheral one, subtending 0.46 and 0.02 deg, respectively, from observer's position. The luminance of the stimuli was measured with a spectroradiometer SpectraScan PR-650 (PhotoResearch, Inc., Chatsworth, CA, USA), values being 175.6 cd/m^2^ for the main stimulus and 61.4 cd/m^2^ for the peripheral one, with the luminance for the background monitor of 0.72 cd/m^2^. The monitor showed 72 peripheral stimuli around the central one, distributed along 18 semiaxes. Each one of the 72 stimuli was presented 2 times. After a 3 min adaptation period to darkness of the monitor background, there was 1 min adaptation to the main stimulus, and then the participant was randomly presented with peripheral stimuli around the central stimulus. The subject, on detecting peripheral spots, pressed a button on the mouse, storing this information for subsequent treatment and calculation of the visual disturbance index (VDI) after the test was finished [6]. The VDI takes values from 0 to 1. The greater value of this parameter indicates that there is a greater contribution of visual disturbances, such as glare, a veil of stray light over the retinal image, or visual halos around luminous stimuli, and therefore poorer discrimination capacity.

In addition to the VDI, the Halo software generates a graph of results where the central stimulus is shown as well as the number of times that each peripheral stimulus is detected by the observer (X for being undetected and 1 or 2 if detected once or twice, resp.), placed in the corresponding position where each stimulus was shown. This graph describes the shape of the visual disturbances perceived by the observer, offering information on areas around a high-luminance stimulus where the observer presents difficulties on detecting luminous stimuli.

Pupil diameter was measured with a Colvard pupillometer (OASIS Medical, Inc. Glendora, CA, USA) before and after alcohol consumption, under the Halo-test illumination conditions.

## 3. Results

Table 2 shows the mean values for the VDI and pupil size before and after alcohol consumption. Comparisons between data were considered to be statistically significant for *P* values of less than 0.05. The VDI was significantly higher after alcohol consumption (*P* < 0.05), indicating an impairment in the visual-discrimination capacity under low-illumination conditions. This impairment was stronger for the group with high alcohol content (BrAC ≥ 0.25 mg/L). In terms of gender, the means of the VDI were of 0.22 ± 0.16 (males) and 0.24 ± 0.14 (females) under normal conditions. After alcohol consumption, the means were of 0.34 ± 0.24 (males) and 0.38 ± 0.22 (females). At the 0.05 level, the means of the VDI were not significantly different between males and females (*P* > 0.05). On the other hand, although the deterioration of the visual-discrimination capacity after the alcoholic intake was similar for both genders (nonsignificant differences), the female group registered a higher mean value (0.114 and 0.147 for males and females, resp.). For the high-alcohol group (BrAC > 0.25 mg/L), the mean VDI deterioration was 0.148 (males) and 0.193 (females), resulting in a higher average deterioration for females.

**Figure 1:**
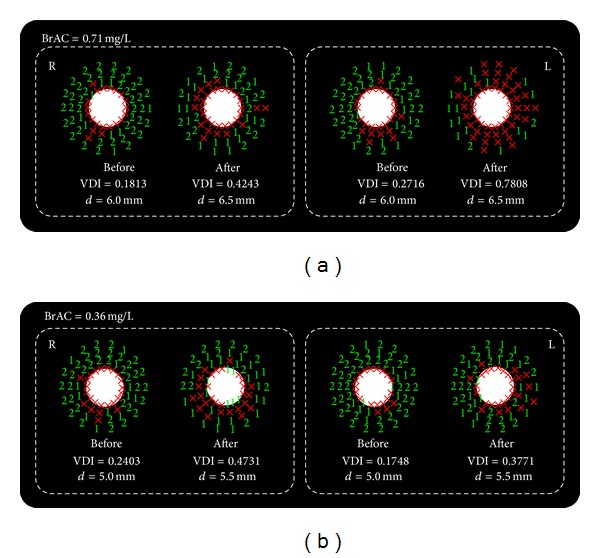
Graphic results for the visual-discrimination capacity (monocular condition) of two participants (breath alcohol contents (BrACs) of 0.36 and 0.71 mg/L) before and after alcohol administration. The corresponding VDI and the pupil size (mm) are included.

**Figure 2:**
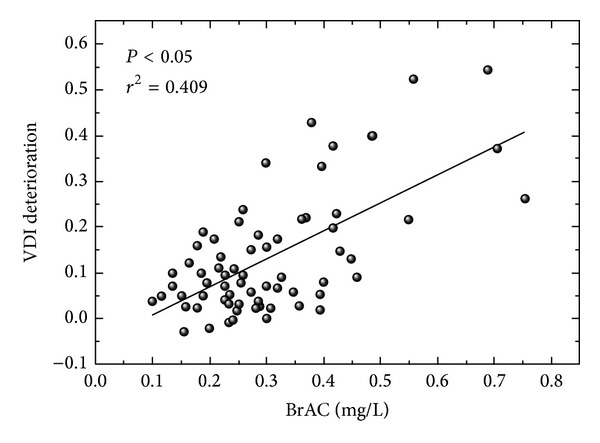
Deterioration for the visual disturbance index (VDI) as a function of breath alcohol content (mg/L).

Figure 1 represents graphic results for two participants (BrACs of 0.36 and 0.71 mg/L) before and after alcohol intake. Under the influence of halos around the main stimulus, a higher VDI value indicates a lower amount of peripheral stimuli detected. The higher the VDI, the higher the halo size around the central luminous stimulus, reducing the visual-discrimination capacity of stimuli. The deterioration of the VDI as a function of alcohol content is represented in Figure 2. For each participant, this deterioration was calculated as the average of the difference between VDI post- and prealcohol intake for each eye. The statistical analysis was performed with a regression analysis from an analysis of variance that provided the *P* value and the *r* ^2^ correlation coefficient. We found a significant ascending correlation (*P* < 0.05, *r* ^2^ = 0.409) for VDI deterioration with BrAC: the higher the BrAC, the greater the deterioration for the visual-discrimination capacity, showing a worsened ability to detect peripheral stimuli around a high-luminance one.

Regarding the pupil size, the results showed a significant increase (*P* < 0.05) in pupil diameter for the high-BrAC group after imbibing alcoholic drinks. Participants with BrAC < 0.25 mg/L did not show significant differences in the pupil size after the alcohol intake.

The results for the optical quality of the eye before and after alcohol intake are presented in Table 3. The average values found for the Strehl ratio under normal conditions were similar to those reported by several authors studying groups of young subjects [20, 21]. Parameters evaluating optical quality deteriorated after alcohol consumption. The Strehl ratio was significantly lower after alcohol intake (*P* < 0.05) in all cases. This decrease indicates a higher level of optical aberrations and intraocular scattering working jointly and therefore poor retinal-image quality. Considering the scattering, the OSI was higher under alcohol effects in all groups, being significantly higher (*P* < 0.05) for participants with BrAC ≥ 0.25 mg/L. The increase in the OSI indicates a greater amount of scattered light through the ocular media and, therefore, retinal-image quality is disturbed after having alcoholic drinks. By gender, before alcohol consumption, the Strehl ratio was of 0.252 ± 0.077 (males) and of 0.247 ± 0.066 (females), with no significant differences (*P* > 0.05) between the two groups. These results agree with those of other authors in a healthy young population [22]. The differences between males and females were not significant, either, after alcohol consumption, registering values of 0.222 ± 0.076 (males) and of 0.220 ± 0.069 (females), respectively. The results for optical quality showed a similar deterioration for males and females after imbibing alcoholic drinks, with no significant differences (*P* > 0.05). The OSI did not significantly vary with gender, as shown by other results [22].

The PRT test gave a mean value of 23.8 ± 7.6 mm before alcohol intake and of 21.6 ± 8.3 mm afterwards for all the participants. The tear-film volume was significantly lower after alcohol consumption (*P* < 0.05) for the group BrAC ≥ 0.25 mg/L. In this case, 71.95% of the eyes fell in tear-film volume. Some authors found no significant changes after moderate alcohol consumption using the Schirmer test [14], agreeing with our results for the low-BrAC group.

**Figure 3:**
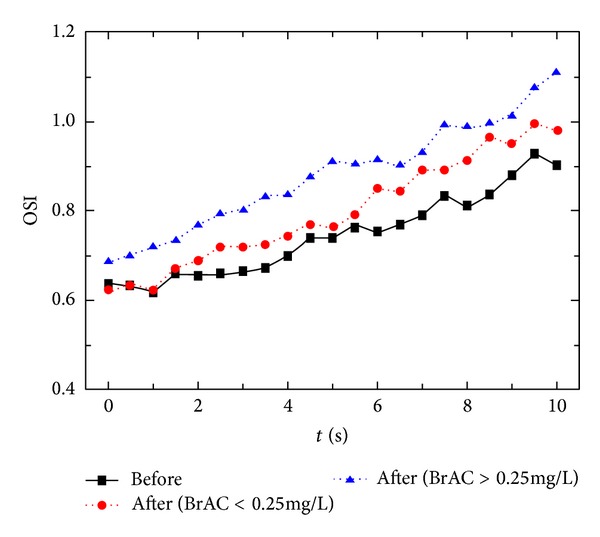
Time course of the optical scattering index (OSI) before alcohol intake (all participants) and afterwards (groups with a breath alcohol content (BrAC) lower or higher than 0.25 mg/L).

Figure 3 presents the time course of the mean OSI before (all participants) and after alcohol intake for the groups with a BrAC lower and higher than 0.25 mg/L. A short time after the blink, the OSI increased with time, due to the deterioration in the tear film in the absence of blinking, which preceded the tear-film break-up. The increase with time of the OSI was more pronounced after alcohol intake, agreeing with results for the OSI (Table 3). For the group BrAC ≥ 0.25 mg/L, the increase after alcohol intake was stronger compared with the low-alcohol-level group, indicating a greater deterioration in the retinal-image quality due to disturbances of the tear film, thereby increasing the scattering through the ocular media and the optical aberrations. The results found here agree with some authors who found a shortened TBUT after alcohol administration [14], resulting in changes in the anterior surface of the eye, which contribute to the reduction in retinal-image quality [23].

## 4. Discussion

The night-vision disturbances perceived by the subjects increased after alcohol consumption, in such as way that the higher the rate of breath alcohol the poorer the visual discrimination capacity. This implies a reduction in the visual-discrimination capacity of peripheral stimuli around the central high-luminance stimulus, therefore deteriorating the visual performance. Under these experimental conditions, the perception of dysphotopsias (halos, starbursts, etc.) is accompanied by a deterioration of the contrast sensitivity [8] and a longer time required to recover it after exposure to a high-luminance stimulus [12]. Age is another major factor in the deterioration of visual performance, in such a way that it also deteriorates contrast sensitivity and visual-discrimination capacity under low-illumination conditions, in agreement with recent results [24], showing the importance of evaluating functions such as the visual discrimination capacity, given that they provide information which is useful to evaluate the visual performance but that alone could not provide other functions such as visual acuity.

Alcohol consumption also can cause physiological changes in the eye, such as the pupil size. Although changes in the pupil diameter after alcohol intake are dynamic and depend on the physiological characteristics of the subject, we found that, for high alcohol content, pupil size increased for most of the subjects. In the literature, some studies reported that chronic alcohol use can adversely cause slight mydriasis [25, 26] or that low and moderate alcohol doses do not affect pupil size [27], whereas some authors found constriction and dilation in pupils, but the limits of such changes were not the same for all subjects [15]. Furthermore, other physiological changes occurred in the eye, due to disturbances of the tear film. Some authors have reported the presence of ethanol in tears after alcohol administration as well a shortened TBUT [15]. Our results indicated a decrease in the tear-film volume using the PRT test. This decrease could be due to the presence of ethanol in tears, especially for subjects with a high alcohol content. This ethanol would disturb the tear-film structure, facilitating the evaporation of the aqueous layer, deteriorating the tear film, shortening the TBUT [15], and diminishing the tear-film volume, as found in the present study. We corroborated these results, demonstrating that scattering through the eye was stronger after alcohol consumption, and, in the absence of blinking, the tear film deteriorated more quickly after alcohol consumption, especially for high-alcohol levels. The increase in scattering and optical aberrations was due largely to the deterioration of the tear film before the break-up, since tear structure was altered, resulting in an uneven film. This could result in small areas of the corneal epithelium being exposed, which is a rough surface compared to the entire tear-film surface, thereby augmenting the wavefront aberrations [28] and scattering and therefore deteriorating the retinal-image quality. Furthermore, our objective data showed a deterioration in the optical quality after alcohol consumption, especially for the high-BrAC group, for which optical aberrations and scattered light through ocular media deteriorated the optical quality of the eye, resulting in a worsened retinal-image. This negative effect depends on the tear film, as discussed, and also on the pupil size, so that the larger the pupil size, the worse the retinal-image quality, because the light penetrates the greater volume of ocular medium, increasing optical aberrations of the eye and intraocular scattering.

With respect to gender, the results for optical quality proved similar for males and females, with no significant differences between groups, as reflected in other studies [22]. The deterioration of this ocular parameter after alcohol intake is not affected by gender, either. For visual performance, there were no differences between genders for the VDI means, nor before or after alcohol intake. On evaluating the deterioration of visual discrimination capacity, we found no significant differences, either, between genders, although such deterioration was higher in the female group, both considering all the participants, as well as in terms of the BrAC classification. These results could be due to the fact that the female group also reached an average BrAC value that was slightly higher than in the male group, although this result would also be consistent with other authors who have found greater alcohol impairment in women in different behavioral tests, such as simulated driving performance, motor coordination, speed of information processing, and information-processing capacity [29]. Although in this work the differences between males and females were not significant, it would be helpful to make broader studies to evaluate such differences, for example, taking into account biometric aspects, such as the BMI (Body Mass Index), and controlling not only for the breath alcohol content of each participant but also for the amount of alcohol consumed.

The present work offers useful results for society and public health, given that it evaluates visual performance and ocular optical-quality before and after alcohol intake. These results could be taken into considerations in such fields as driving, where it is of vital importance to evaluate the visual abilities of the subject, especially under night-time conditions, where physiological changes and visual alterations occur. Our results show that the parameters and visual test performed here could be used to evaluate the visual state of subjects, providing useful information for evaluating the visual capacities, a very important aspect in getting and renewing a driver's license, where visual functions such as visual acuity do not completely evaluate the visual state of the subject.

## Figures and Tables

**Table 1:** Classification of the participants based on the breath alcohol content (BrAC) limit. The average values of the BrAC (mg/L) are shown for each group. Standard deviation included. Classification of the subjects according to gender is also shown.

	BrAC < 0.25 mg/L	BrAC ≥ 0.25 mg/L	Total
Participants			
Males	12	20	32
Females	14	21	35
All	**26**	**41**	**67**
BrAC (mg/L)			
Males	0.18 ± 0.04	0.37 ± 0.11	0.30 ± 0.13
Females	0.20 ± 0.04	0.39 ± 0.14	0.31 ± 0.14
All	**0.19 ± 0.04**	**0.38 ± 0.13**	**0.30 ± 0.14**

**Table 2:** Mean values for the visual-disturbance index (VDI) and the pupil size, before and after alcohol consumption for all participants and participants with a BrAC greater or less than 0.25 mg/L. Standard deviations included.

BrAC (mg/L)	<0.25	≥0.25	Total
VDI			
Before	0.19 ± 0.09	0.25 ± 0.17	0.23 ± 0.15
After	0.26 ± 0.14	0.42 ± 0.25	0.36 ± 0.23
*P* value	<0.001	<0.001	<0.001
Pupil size (mm)			
Before	5.14 ± 1.05	5.24 ± 0.90	5.20 ± 0.96
After	5.15 ± 0.99	5.70 ± 0.93	5.49 ± 0.99
*P* value	0.452	<0.001	<0.001

**Table 3:** Mean values for the optical-quality parameters of the eye, before and after alcohol intake, for all the participants and for the groups of high and low alcohol levels (breath alcohol content [BrAC] in mg/L). Strehl ratio, MTF (modulation-transfer function) cut-off, optical scattering index (OSI), and tear volume (in mm) are presented. Standard deviation included.

BrAC (mg/L)	<0.25	≥0.25	Total
Strehl ratio			
Before	0.248 ± 0.068	0.251 ± 0.074	0.249 ± 0.072
After	0.232 ± 0.072	0.214 ± 0.071	0.221 ± 0.072
*P* value	0.023	<0.001	<0.001
OSI			
Before	0.55 ± 0.35	0.56 ± 0.45	0.56 ± 0.41
After	0.60 ± 0.56	0.73 ± 0.61	0.68 ± 0.59
*P* value	0.184	<0.001	<0.001
Tear volume (mm)			
Before	23.2 ± 7.3	24.3 ± 7.8	23.8 ± 7.6
After	22.2 ± 9.2	21.3 ± 7.7	21.6 ± 8.3
*P* value	0.476	0.001	0.003
